# Assessment of Frozen Stored Silver Carp Surimi Gel Quality Using Synthetic Data-Driven Machine Learning (SDDML) Model

**DOI:** 10.3390/gels11100810

**Published:** 2025-10-09

**Authors:** Jingyi Yang, Shuairan Chen, Tianjian Tong, Chenxu Yu

**Affiliations:** 1 Department of Agricultural and Biosystems Engineering, Iowa State University, Ames, IA 50011, USA; jingyiy@iastate.edu; 2 Department of Computer Science, Iowa State University, Ames, IA 50011, USA; shuairan@iastate.edu; 3 Department of Chemical and Biological Engineering, Iowa State University, Ames, IA 50011, USA; tianjian@iastate.edu

**Keywords:** frozen effect, silver carp, surimi gel, manufactured microfiber, transglutaminase, collagen, data augmentation, synthetic data generation, random forest model

## Abstract

The invasive Silver Carp (*Hypophthalmichthys molitrix*) in North America represents a promising resource for surimi production; however, its gel formability deteriorates significantly during frozen storage. This study investigated the deterioration of gel properties in Silver Carp surimi over six months of frozen storage, and showed that short-term frozen storage (<2 months) was beneficial for surimi gel-forming ability, while extended frozen storage (>2 months) tended to have detrimental effects. The adverse effect of long-term frozen storage could be mitigated via using food additives (e.g., manufactured microfiber, transglutaminase, and chicken skin collagen), among which transglutaminase was the most effective. Transglutaminase at a relatively low level (0.1 wt%) could effectively negate frozen storage’s effects, and produced surimi gel with quality attributes (e.g., gel strength, hardness, and chewiness) at levels comparable to those from fresh fish samples. To assess the effects of the addition of various food additives for quality improvement, a synthetic data-driven machine learning (SDDML) approach was developed. After testing multiple algorithms, the random forest model was shown to yield synthetic data points that represented experimental data characteristics the best (R^2^ values of 0.871–0.889). It also produced improved predictions for gel quality attributes from control variables (i.e., additive levels) compared to using experimental data alone, showing the potential to overcome data scarcity issues when only limited experimental data are available for ML models. A synthetic dataset of 240 data points was shown to supplement the experimental dataset (60 points) well for assessment of the Frozen Silver Carp (FSC) surimi gel quality attributes. The SDDML method could be used to find optimal recipes for generating additive profiles to counteract the adverse effects of frozen storage and to improve surimi gel quality to upgrade underutilized invasive species to value-added food products.

## 1. Introduction

Silver Carp (*Hypophthalmichthys molitrix*), one of the Asian Carps, is threatening the ecosystem of North America as an invasive species [1]. The utilization of the Asian Carp as a food resource remains limited because they are foreign to the American diet. To promote harvesting of these invasive fish, market-welcomed products are needed to produce strong economic incentives. Surimi gel is a fully cooked, protein-rich food made with fish mince through thermal gelation, which could provide a pathway for the marketing and utilization of silver carp. Surimi gel is very popular in East Asian countries and has gained increasing appeal in the European and North American markets over the last decade. Surimi gel is usually made with marine fish, and the overfishing and depletion of marine fish species are threatening the health of the surimi gel industry. Utilization of freshwater invasive fish such as Silver Carp (SC) could ameliorate this problem, reduce the cost of surimi gel production, and at the same time generate a commercial market for the fish with increased customer interest.

However, SC performs less well in surimi gelation. Surimi gel is formed when the myofibrillar proteins (about 70% of total protein in fish muscle) are aggregated and cross-linked through a thermal process [2,3]. The myofibrillar proteins of SC, especially myosin (about 60% of total myofibrillar proteins), are susceptible to the “modori” phenomenon, which causes gel degradation during the thermal process due to the breakdown of myosin by endogenous proteases [4,5]. The freshness of the fish is also a critical point for surimi gel products. The frozen storage process to extend fish shelf-life could lead to unexpected myofibrillar protein denaturation, aggregation, chemical modification, and loss of water-holding capacity (WHC), which causes poor surimi gel quality [6,7,8,9,10,11,12].

Protein denaturation during frozen storage is a detrimental process that contrasts with controlled thermal gelation. Unlike the reversible, ordered unfolding that forms a strong elastic gel network during heating [6], freezing causes irreversible damage [7]. The synergistic effect of ice crystallization and low temperature disrupts the protein’s secondary and tertiary structures [8]. This random unfolding exposes hydrophobic residues, leading to disordered and irreversible aggregation that prevents effective gel formation.

Furthermore, freezing induces harmful chemical modifications. It generates reactive oxygen species that oxidize amino acids, impairing crucial disulfide bond formation for crosslinking [9,10]. It also disrupts cells, releasing endogenous enzymes that proteolyze myofibrillar proteins, further diminishing gel-forming capacity [11].

This collective denaturation, aggregation, and modification cause myofibrillar proteins to lose their native function, resulting in a severe loss of water-holding capacity (WHC) [12]. Consequently, premium surimi products rely on fresh fish, making them expensive. This creates a high demand for remedies, such as surimi from FSC, that can mitigate these freeze-induced defects and improve gel quality, enhancing their economic viability.

The duration of the frozen storage also impacts the surimi gel quality. Research on four commonly used surimi fish in Thailand (i.e., threadfin bream, bigeye snapper, lizardfish, and croaker) stored at −18 °C for a 6-month period found a declining trend in gel-forming ability due to protein denaturation, and the degree of the deterioration in gel-forming ability was dependent upon the species and frozen time [13]. Research on SC captured in China stored at −18 °C for a 3-month period similarly revealed that the gel strength decreased along with the frozen time [14]. Although the native SC in China and the invasive SC in North America are the same species, research has found that significant genetic differences exist between the SC population in China (Yangtze, Pearl, and Amur Rivers) and the population in North America (Mississippi River) [15]. The differences in diet and growth conditions also influence the protein characteristics of fish [16,17]. Therefore, a study using SC captured in North America is necessary to understand the effects of frozen storage on surimi gel formation of the SC surimi in North America. In addition, research on Herring fish revealed that frozen time and pH could affect the rheological properties of Herring fish protein [18]. However, there are few studies on the effects of frozen storage time on the rheological properties of SC surimi paste. Understanding frozen-storage effects on SC surimi rheology can guide its utilization as food ink for 3D printing-based innovative food production, which can lead to value-added food production to further enhance the economic incentives for its utilization.

Our previous study found that additives such as manufactured microfiber (MMF), transglutaminase (TG), and chicken collagen (CLG) can improve the gel strength, texture properties, WHC, and gel microstructures of frozen-stored SC (FSC) surimi gel [19]. MMF is a cellulosic fiber with a length of a few micrometers and a width of a few hundred nanometers, serving as a potent reinforcing agent for protein crosslinking with a high aspect ratio, superior mechanical strength, and a high hydroxyl group density [20]. TG is broadly used in the food industry as meat glue since it can catalyze surimi gel cross-linking with ε-(γ-glutamyl) lysine bonds [21]. CLG is a functional protein that enriches the amino acids to form more protein crosslinking during thermal gel formation [22]. A Response-Surface Modeling (RSM) was developed in the previous study for process optimization to find the best MMF, TG, and CLG combination for improving FSC surimi gel, with limited success due to the complexity of the problem [19]. Recent research indicated that RSM’s fixed-order polynomial regression performed less well than the more accurate and flexible Machine Learning (ML) models [23]. Therefore, ML-based models are explored in this study to better elucidate how additives may be used to counteract FSC surimi’s poor gelation performance.

However, the construction of effective machine learning models can be greatly restricted due to the lack of sufficient data. Preparation of surimi gel samples out of FSC is time- and cost-consuming. In our previous report, we had only four replicated sample groups with 15 treatments (i.e., three levels of each of the three additives, with different combinations in a Box–Behnken experimental design [19]) for each group, which yielded 60 data points in total. The sparsity of the available data was part of the reason why the RSM model did not work very well. The scarcity of data in a small dataset could cause issues such as overfitting and high noise for ML models as well [24]. As physical constraints always exist in most experimental data acquisition work, data augmentation techniques such as data synthesis have been explored as a means to mitigate data scarcity issues related to small-dataset limitations [25]. In this study, we aimed to improve an ML-driven model for FSC surimi gel optimization using data synthesis to supplement the experimental dataset. Various regression approaches, including Linear Regression, Polynomial Regression, Ridge Regression, Support Vector Regression (SVR), Decision Tree, Random Forest, Gradient Boosting, Bayesian Ridge, Automatic Relevance Determination for Bayesian Ridge (ARD), Multilayer Perceptron (MLP), and Extreme Gradient Boosting (XGB) were investigated as means to generate synthetic dataset which could mimic the original experimental data, and the one with the best performance (i.e., highest coefficient of determination R^2^) was used to generate synthesized dataset. Kernel Density Estimation (KDE) was employed to generate the synthesized data based on the original experimental data because it performed better on small datasets [26]. The synthesized data were screened with the constraints of showing statistical characteristics mimicking the original dataset until a pre-determined size of dataset (i.e., 120, 180 and 240 data points) was reached, and the synthesized data were then pooled with the original experimental data to train ML models for the assessment and optimization of FSC surimi gel properties with different valuation of control variables (i.e., levels of additives).

In addition, in this study, the effects of frozen storage time on surimi gel quality for SC captured in North America was evaluated by gel strength, texture profile analysis (TPA) properties (e.g., hardness, springiness, and cohesiveness), rheological properties (via the small-amplitude oscillatory shear method (SAOS) and hysteresis loop test), expressible moisture (EM), and cooking loss. A Synthetic Data-Driven ML (SDDML) model was developed to predict the gel properties from additive dosage, and ultimately, determine the optimal additive combinations for achieving the desired gel properties tailored towards applications such as 3D printing food ink. The methods created in this research could be easily expanded to include other quality attributes and could be adaptable for other Asian Carp and/or fish species, providing a powerful tool for developing ML-driven predictive models for product optimization.

## 2. Results and Discussion

### 2.1. Gel Strength and Textural Properties

**Figure 1:**
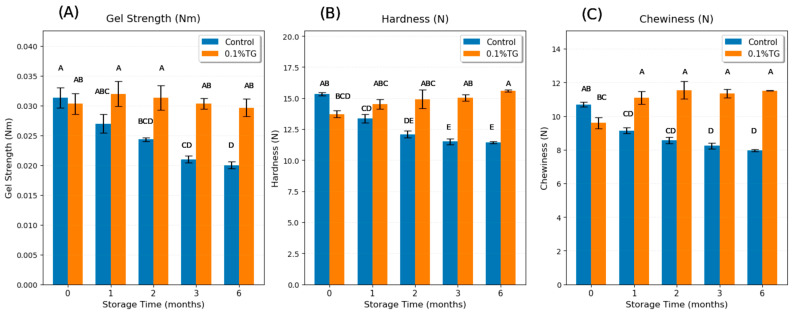
Gel strength (**A**), hardness (**B**), and chewiness (**C**) results of control FSC surimi samples (blue) and 0.1 wt% TG-added FSC surimi samples (orange) along with frozen storage time. Uppercase letters on error bars show the significant differences (*p* < 0.05).

Gel strength, defined as the multiplication of breaking force and deformation distance [27], is one of the important parameters to evaluate surimi gel quality and the degree of protein denaturation [14]. Figure 1A shows a significant decrease (*p* < 0.05) in gel strength of control FSC surimi samples from fresh to 6-month frozen. There was a sharp decline from fresh to 2-month frozen, and it became relatively stable from 2-month frozen up to 6-month frozen. These results are consistent with the findings in SC captured in China [14] and threadfin bream, bigeye snapper, and croaker captured in Thailand [13], which also showed a noticeable decrease within the first 2 months frozen and followed by stabilization. The gel strength of the control samples decreased by 36.2% from fresh to 6-month frozen. The data for the 4th and 5th months of frozen storage were not reported as the 3-month time point was deemed a functionally critical threshold, demonstrating severe irreversible protein denaturation [13,14]. The experimental design focused on capturing the progression of quality loss during the active degradation phase (0–3 months) and a final endpoint representing extreme storage conditions (6 months). Indeed, no significant changes were observed in samples of 4- and 5-month frozen storage from those of either 3 months frozen storage or 6 months frozen storage.

The reduction in gel strength showed that the frozen storage progressively weakens the gel formability of FSC due to protein denaturation and loss of cross-linking ability [7,8]. The one-month frozen control sample showed no significant difference from the fresh sample because at this point, the proteins were just partially denatured. They had begun to lose their structural integrity, but were not completely unfolded and aggregated yet. After 2 months of frozen storage, more proteins had become partially or fully denatured, and after 6 months, a large portion of the proteins were fully denatured, and their gelation performance was significantly deteriorated. In contrast, the 0.1 wt% TG-added samples maintain stable gel strength along with frozen time, remaining statistically higher than the control samples after 2–6 months. Notably, there was no significant difference between 0.1 wt% TG-added samples for all frozen periods and the fresh control sample, suggesting that 0.1 wt% TG enzyme is sufficient to mitigate the adverse frozen impacts of SC on gel strength. TG enzyme catalyzes the formation of covalent ε-(γ-glutamyl) lysine bonds, thereby restoring the cross-linking ability damaged by frozen storage [21,27]. The other two additives (MMF and CLG) were also investigated at various levels to assess their abilities to negate the adverse effects of frozen storage at the 1-month time point. None showed any statistically significant effects on the quality attributes of the gel (e.g., gel strength, hardness, chewiness, etc.); hence, their effects were not further investigated. Consequently, additives such as microfiber (MMF) and collagen (CLG) were ineffective at negating these adverse effects. This is because their mode of action is that of an external filler rather than a functionally restorative for the denatured protein network. While MMF provides an extra physical network, especially for water retention [20], and CLG forms a kind of weak gelatin gel separately [22], neither additive can reverse the underlying protein denaturation or integrate well with the damaged proteins to restore the quality attributes.

Textural properties, including hardness, cohesiveness, springiness, resilience, and chewiness, were measured by Texture Profile Analysis (TPA) to simulate the chewing process of the surimi gel [28]. Hardness measures the force needed to fracture the gel. Cohesiveness indicates the gel’s ability to maintain its integrity during a second compression. Springiness is the capacity to return to its original shape after deformation, following a brief delay. In contrast, resilience is the immediate capacity to recover its height after deformation, without any delay. The product of hardness, cohesiveness, and springiness results in chewiness, which indicates how easily the surimi gel can be chewed [29].

Figure 1B shows a sharp decline in the hardness of the control FSC surimi gel from fresh to 2-month frozen, after which it remained relatively stable. Figure 1C indicates a rapid decrease from fresh to 1-month frozen and remained relatively stable afterward. Overall, hardness decreased by 25.5% and chewiness decreased by 25.6% from fresh to 6-month frozen, likely due to protein denaturation and loss of cross-linking ability during the frozen storage [7,8]. The decline in gel properties of the control samples from fresh to 2-month frozen storage showed that the protein denaturation was partial and in progress, which then stabilized after 2 months, suggesting that enough protein denaturation had occurred by 2 months; further frozen storage did not bring significant changes after that. In contrast, the 0.1 wt% TG-added FSC surimi samples had no significant differences from the fresh control sample, suggesting that 0.1 wt% TG was sufficient to counteract the adverse frozen impacts on hardness and chewiness. The results of hardness and chewiness had similar trends with gel strength because hardness and chewiness are generally consistent with gel strength, indicating the integrity of the 3-D gel network [27].

Table 1 lists the remaining TPA results, including springiness, cohesiveness, and resilience. No significant difference was observed in springiness, indicating that the frozen effect on springiness is too minor to be detected by TPA. Similarly, there was no significant difference in the cohesiveness readings among the samples, suggesting that frozen storage did not substantially impair the ability of the gel to maintain its structural integrity during deformation. In contrast, the 0.1 wt% TG-added samples exhibited higher cohesiveness than the control samples because TG enzyme promoted the formation of covalent ε-(γ-glutamyl) lysine bonds, strengthening the integrity of the 3-D gel network [21,27]. Interestingly, the resilience of the control samples sharply increased from fresh to 1-month frozen and then gradually decreased. This is consistent with the findings that short-term frozen storage can temporarily enhance the texture properties through partial protein denaturation and aggregation [30]. When a small or controlled amount of protein denaturation occurred, it acted as a beneficial “pre-cooking” step, leading to mild unfolding that exposed reactive parts of the protein (like hydrophobic groups and sulfhydryl groups) that are normally buried inside the folded structure. However, when denaturation went beyond a certain threshold, it became destructive, breaking down the protein’s ability to form a gel at all. In our study, this threshold for frozen storage of silver carp was shown to be 2 months of storage time. In comparison, the 0.1 wt% TG-added samples maintained higher resilience than the control, indicating that TG-enhanced cross-linking improved the gel’s ability to recover its structure after deformation [21,27].

### 2.2. Cooking Loss and Expressible Moisture

**Figure 2:**
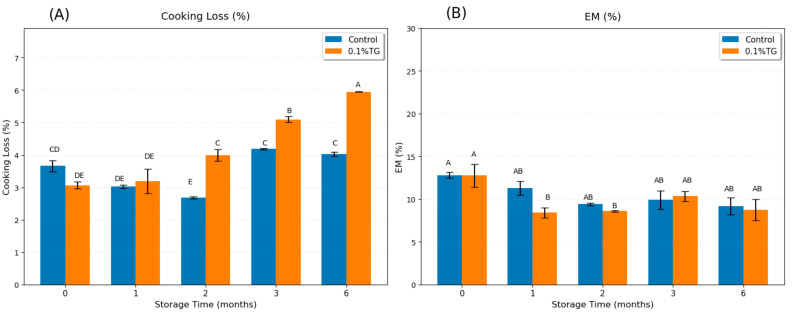
Cooking Loss (**A**) and EM (**B**) results of control FSC surimi samples (blue) and 0.1 wt% TG-added FSC surimi samples (orange) along with frozen storage time. Uppercase letters on error bars show the significant differences (*p* < 0.05).

Cooking loss is the loss of liquid and soluble matter percentage during heating, mainly from water loss [31]. A denser and more stable network of surimi gel can retain more water (i.e., higher WHC) [30], resulting in lower cooking loss. In Figure 2A, the cooking loss of control samples is shown to sharply decline in the first 2 months of frozen storage, and then return to the level of the fresh control samples, consistent with the findings that short-term frozen storage can temporarily increase WHC by partial protein denaturation and aggregation [30]. These limited changes exposed hydrophilic groups and hydrophobic patches, prompting the formation of a temporary network to effectively entrap water, leading to a transient rise in WHC [30]. The cooking loss of 0.1 wt% TG-added samples increased rapidly after the first month of frozen storage. Two factors may explain this: (i) the WHC decreased during long-term frozen storage [14,30], though the minor change in the control samples after 3 months frozen storage suggested this effect might be quite limited; and (ii) the excessive TG cross-linking reduced protein–water interactions, thereby reducing WHC [32]. The results suggested that 0.1 wt% TG was effective when proteins were still relatively intact (after short-term frozen, <2 months), but became excessive in long-term frozen storage (>2 months storage), where denatured proteins became less able to retain water.

Expressible moisture (EM) is defined as the percentage of loose water released under force relative to total moisture and is commonly used to evaluate WHC [13]. A high EM indicates a poor ability to retain water (i.e., low WHC). As shown in Figure 2B, no significant difference was observed among the control samples over frozen storage up to six months, nor between the control and the 0.1 wt% TG-added samples at the same frozen period. This suggested that the frozen time effect on EM was too minor to be detected for WHC evaluation of control samples. A significant decrease in EM was observed in samples with 0.1 wt% TG after short-term frozen storage (<2 months), indicating an improvement in WHC. This aligned with previous findings, suggesting this TG concentration was effective when the frozen-caused partial protein denaturation and aggregation were overcome. However, its efficacy diminished with long-term storage (>2 months), as extensive protein denaturation reduced the substrate availability and functionality necessary for TG to form a well-hydrated gel network.

### 2.3. Rheological Properties

#### 2.3.1. Small-Amplitude Oscillatory Shear (SAOS) Test

**Figure 3:**
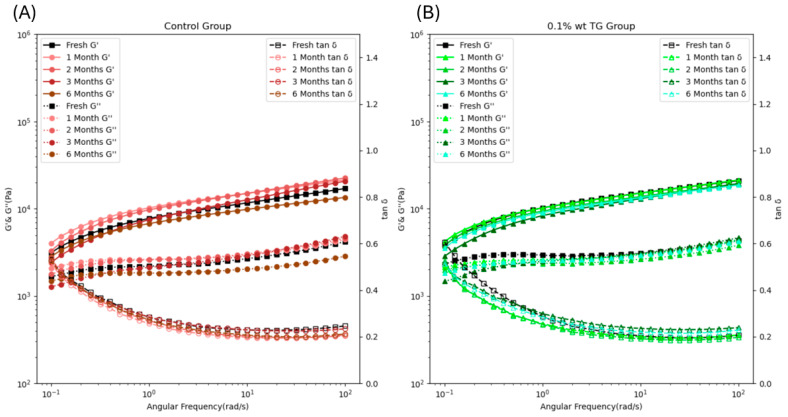
The storage modulus (G′) (solid line with solid marker), loss modulus (G″) (dashed line with solid marker), and the tan б (dashed line with hollow marker) of the control FSC paste samples (**A**) and 0.1 wt% TG-added FSC paste samples (**B**) along with frozen storage time.

The SAOS test was conducted to measure the storage modulus (G′), loss modulus (G″), and the tan б, which were used to determine the relationship between elastic (solid-like) and viscous (liquid-like) behavior. In Figure 3, the G′s of both the control and 0.1 wt% TG-added samples were consistently greater than the corresponding G″s (i.e., tan б < 1), demonstrating solid-like behavior of well-formed gel [33]. Furthermore, the G′ and G″ were parallel and gradually increasing, indicating a strong gel network [33], which would benefit 3-D printing of the fish paste as a food ink to maintain its shape and ensure consistent extrusion flow during 3-D printing [34]. In Figure 3A, an increase in storage modulus (G′) of the control FSC paste was observed from fresh to 2-month frozen storage, and declined thereafter, with a notable reduction at 6 months. These results aligned with the findings that a short-term freeze can temporarily enhance the gel network [30], and the extended frozen time damages the protein structure, and decreases both G′ and G″. In contrast, the viscoelastic properties of the 0.1 wt% TG-added sample, as shown in Figure 3B, were relatively stable, suggesting that the TG enzyme can effectively mitigate the frozen effects on viscoelastic properties. The TG enzyme counteracted the damage of frozen storage by forming strong covalent ε-(γ-glutamyl) lysine bonds. This restored the proteins’ cross-linking ability, creating a stable gel network with robust viscoelastic properties [21,27].

#### 2.3.2. Hysteresis Loop Assessment

**Figure 4:**
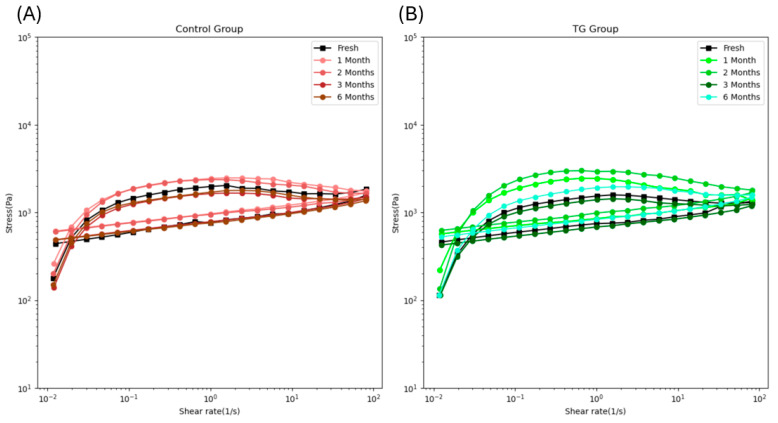
The thixotropic loop of the control FSC paste samples (**A**) and 0.1 wt% TG-added FSC paste samples (**B**) along with frozen storage time.

Thixotropic behavior refers to the ability of a material to decrease in viscosity under applied shear stress and gradually recover when the stress is removed, which is an important property to evaluate the 3-D printability of a food ink [33]. Viscosity deduction during extrusion for 3-D printing allows the surimi paste to flow smoothly through the nozzle, while viscosity recovery after the extrusion would be instrumental in maintaining the printed shape and preventing its collapse. Hence, the samples with strong thixotropic behavior would be favored as food ink for 3D printing. Figure 4 showed that all samples exhibited thixotropic loops, indicating that all of them had thixotropic behavior. The degree of thixotropy is reflected by the area of the thixotropic loop [33]. Figure 4 shows a notable increase in loop area from fresh to 2 months of frozen storage for both the control and 0.1 wt% TG-added samples, which then returned to the level of the fresh sample after extended frozen storage. The TG-added samples exhibited a larger area in short-term frozen storage than the control samples, indicating that the short-term frozen storage and 0.1 wt% TG addition can improve the thixotropy of FSC paste, thereby benefiting its 3-D printability.

**Figure 5:**
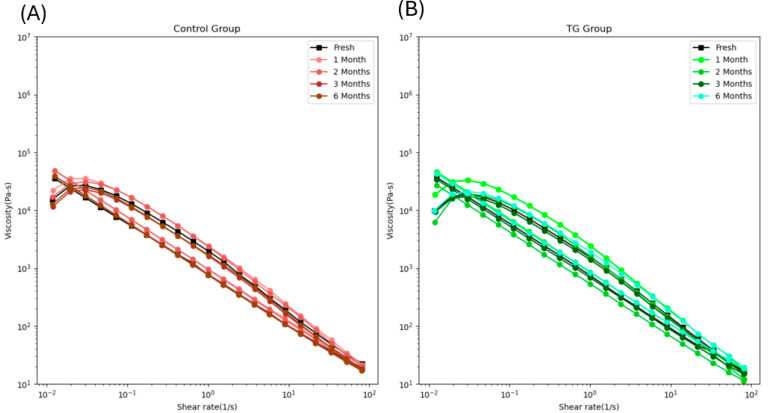
The viscosity loop of the control FSC paste samples (**A**) and 0.1 wt% TG-added FSC paste samples (**B**) along with frozen storage time.

Shear-thinning behavior refers to decreased viscosity with increasing shear rate [33]. In 3D printing, viscosity plays a critical role: if the food ink is too viscous, it cannot pass smoothly through the nozzle, whereas the liquid-like food with low viscosity fails to maintain its printed shape. Therefore, selecting a suitable viscosity range is essential for 3D printing operations. Figure 5 shows that all samples exhibited shear-thinning behavior once the shear rate exceeded 0.025 s^−1^. At the same shear rate, the viscosity of the control samples increased from fresh to 2-month frozen storage, then dropped back after extended frozen storage (Figure 5A). In Figure 5B, the viscosity of the 0.1 wt% TG-added samples increased from fresh to 1-month frozen storage, then returned to the fresh level. These results are consistent with the finding that short-term frozen storage can increase the viscosity of myofibrillar protein [30], thereby improving control of FSC paste viscosity for use as food ink.

Overall, our findings indicated that among the three additives investigated, only TG was truly effective in terms of negating the adverse effects of long-term (>2 months) frozen storage on the gelation properties of the FSC. However, as indicated in our previous work [19], MMF and CLG could improve surimi gel properties when used in synergy with TG. To tailor the FSC gel properties to meet specific needs, such as 3D printing food ink, optimal recipes of additives need to be created. Such a task of optimization requires the ability to make sound assessments and/or predictions of quality attributes based on the compositional control variables (i.e., additive levels), which calls for reliable predictive models. Machine learning algorithms have been reported to provide better predictions than the RSM method [23,24]. In this study, we aimed to explore ML modeling for the assessment of FSC gel qualities. The first task was to explore data synthesis to expand the limited experimental dataset we had at hand, and then to select the best ML models to assess the FSC gel properties based on control variables.

### 2.4. Synthetic Data-Driven Machine Learning (SDDML) Models

#### 2.4.1. Identify the Appropriate ML Model for Data Synthesis

The key to successfully creating synthesized data that could represent the characteristics of the experimental dataset is to identify the statistical characteristics of the experimental dataset that can be applied to the synthesized data. In this study, three common quality attributes used to evaluate gel quality (e.g., gel strength, hardness, and chewiness) were used as the basis to evaluate the characteristics of the experimental dataset. Various popular ML methods were tested using the normalized original data, with the aim of correlating the controlled variables (e.g., levels of additives of MMF, TG, and CLG) to the three quality attributes. The corresponding determination coefficients (R^2^) obtained with the validation dataset are shown in Table 2 for various ML methods investigated. Generally, methods based on linear regression did not perform well for predicting the quality attributes from the controlled variables, evidenced by the consistently lower R^2^, suggesting that simple linear regression cannot capture the complexity of the dataset. In comparison, multilayer perception (MLP) showed a relatively low mean R^2^ with occasionally higher best-case values, which is reasonable because this method is sensitive to the dataset size (60 data points is too small) [35]. Polynomial and ridge regression methods performed better than the linear model but were outperformed by the tree-based ensemble methods (Random Forest, Gradient Boosting, and Extreme Gradient Boosting (XGB)). Among them, the random forest model achieved the highest mean of R^2^ values for the three quality attributes (0.464 for gel strength, 0.605 for hardness, and 0.617 for chewiness). The best-case values of R^2^ for the random forest model were also relatively higher, indicating that the random forest method was the most appropriate model for capturing the characteristics of this dataset. Hence, it was chosen as the method for data synthesis.

#### 2.4.2. Synthetic Data Generation and Validation

**Figure 6:**
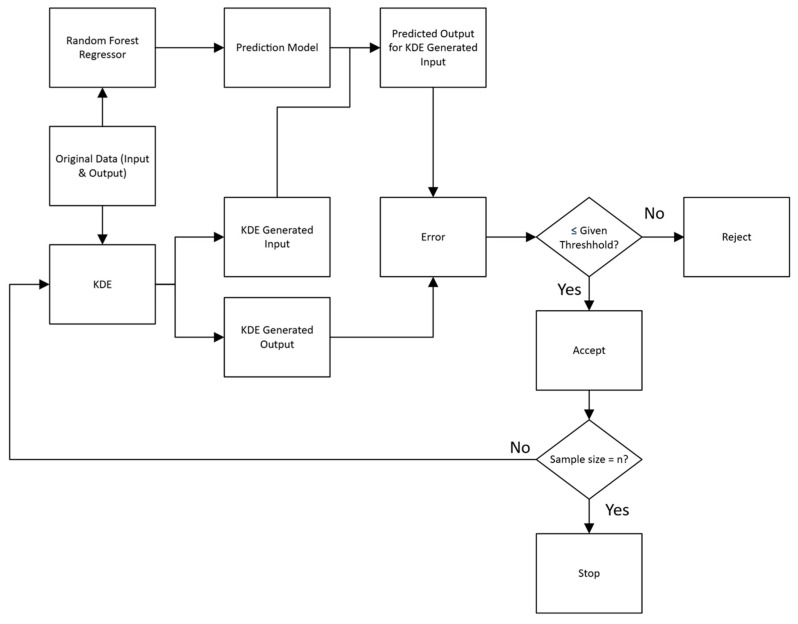
The flowchart of the SDDML method for data generation.

**Figure 7:**
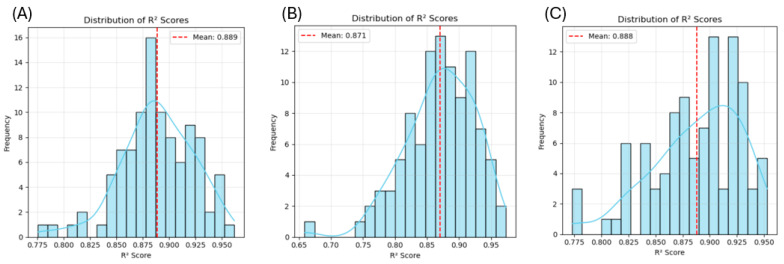
The distribution of R^2^ for the Random Forest model with the mixed dataset of gel strength (**A**), hardness (**B**), and chewiness (**C**).

Figure 6 shows the process of synthetic data generation for our SDDML method. The details of the process were described in Section 4 below. The time needed for the synthetic data generation function to generate 240 synthetic data points was 1 to 2 min. The Random Forest model trained on the generated dataset and tested on experimental data points achieved relatively high R^2^ scores for gel strength (0.702), hardness (0.805), and chewiness (0.831), indicating that the generated synthetic data represented the original experimental data well. Then the generated and original data were mixed and split randomly (240 training and 60 testing) to find the R^2^ distribution of the Random Forest model (Figure 7). The mean R^2^ was further improved, with 0.889 for gel strength, 0.871 for hardness, and 0.888 for chewiness. These results suggested that the synthetic data generated by the SDDML method not only preserved the intrinsic characteristics of the original experimental dataset but also enhanced model robustness and predictive accuracy when combined with real data.

However, it should be noted that the improvement over model prediction accuracy by synthetic data was not limitless. There was no new information carried by the synthetic data, as they were created to represent the characteristics of the experimental data. Their function was to highlight what was already in the experimental data to assist training of the ML algorithm, not to add any new features. Therefore, when synthetic data were combined with the experimental data, the prediction accuracy of the SDDML model was better than that of the ML model of experimental data alone, but not by a lot. To test the prediction accuracy more directly, 18 experimental data points were randomly selected as a testing set, and then the remaining 42 experimental data points were pooled with various numbers of synthetic data points to generate the training dataset for the random forest ML model. The predicted values of the three quality attributes for the testing set were compared to the actual values. The average determination coefficients (R^2^) out of 100 repetitions (each repetition with a randomly selected 18 data point testing set) were compared. As shown in Table 3, the prediction accuracy was improved, evidenced by increases in R^2^ values as well as decreases in the margins of errors when the size of the synthetic dataset increased, significantly for hardness and chewiness, but not significantly for gel strength, even when the size of the synthetic dataset went beyond 1200. Furthermore, the improvement of R^2^ values over ML with only experimental data was not very large, especially for gel strength (~3%). The improvements over the prediction of textual properties (hardness and chewiness) were larger (~7% and 10%, respectively) in comparison. This is reasonable because the prediction for gel strength had the lowest accuracy to begin with for the original experimental data; meanwhile, the Random Forest model performed better at predicting hardness and chewiness values, leading to a larger improvement with the synthetic data from the SDDML method. The synthetic data would provide better training for the SDDML model, hence improving the prediction accuracy. However, they would not provide new information that was not contained in the experimental dataset, which is its limitation.

The less-than-impressive improvement of prediction accuracy of FSC quality attributes out of the control variables (i.e., additive levels) could be attributed to the complexity of the correlations between them. It is reasoned that changes in control variables led to changes in network crosslinking patterns (i.e., microstructures) in the FSC surimi gel formed, which in turn led to changes in quality attributes. Therefore, when efforts were made to predict the quality attributes out of the control variables directly, the complex intermediate process of crosslinking formation was bypassed, and the prediction accuracy, hence, was intrinsically compromised to a certain degree. This obstacle could be overcome by constructing multi-stage ML models corresponding to the intermediate steps: a first-layer ML model to correlate control variables to attributes of the crosslinking network of the FSC gel, then a second-layer ML model to correlate the microstructural parameters to the quality attributes. At each layer, more direct connections from variables to predictors would yield a better assessment of the quality attributes in the end. Work is underway to pursue this approach, and the data synthesis approach developed in this study will be utilized to improve ML assessment at each layer.

## 3. Conclusions

This study investigated the effects of frozen storage on the gel quality of surimi prepared from invasive SC in North America and explored remedies using food additives with the SDDML approach. Extended frozen storage significantly reduced gel strength, hardness, and chewiness due to protein denaturation and aggregation, while short-term frozen storage could be beneficial. The addition of 0.1 wt% TG could effectively negate the detrimental effects of extended frozen storage and brought the key texture properties to levels comparable to those of fresh fish samples, demonstrating its potential as a simple and effective mitigation strategy for frozen storage. However, the high cost of TG and the growing demand for nutrition-enhanced products call for the use of combined additives to gain more economic and quality advantages. As promising alternatives, CLG and MMF were combined with TG in our previous study to obtain higher WHC and better textural properties [19]. To find the best recipe of CLG + MMF + TG, assessment tools to predict quality attributes for surimi gel from control variables are needed. As the complex additive interaction patterns are difficult to predict from first principles, machine learning models were explored in this study as possible solutions. Random forest algorithm was shown to be the most reliable predictive assessment tool for the FSC gel. A data synthesis method was developed to supplement the limited experimental data to provide better assessment performance. The proposed SDDML method successfully generated synthetic data that closely represented the original dataset and enhanced model prediction accuracy when combined with experimental data. This approach offered a pathway to address the small dataset limitation common in food science and provided a robust and accessible framework of synthetic data-driven predictive modeling for non-experts. Ultimately, the SDDML approach offered a promising tool to guide the optimization of additive profiles to overcome the negative impacts of long-term frozen storage on surimi gel quality and improve the control over surimi gel production to generate products to meet specific needs. These findings not only supported the broader utilization of invasive Silver Carp in value-added food production but also established an adaptable methodological framework for other food science applications.

## 4. Materials and Methods

### 4.1. Materials

Silver Carp fillets (with skin and bones) were obtained from Two Rivers Fisheries, Inc. in Wickliffe, KY, USA. Manufactured microfiber (MMF) was supplied by the University of Maine, Orono, ME, USA. A food-grade TG (100–120 U/g activity), selected for its economic viability and common use in food applications, was purchased from Modernist Pantry, Eliot, ME, USA. Chicken collagen (CLG) was a product of Essentia Protein Solutions, Ankeny, IA, USA. The Sodium chloride, NaCl, was bought from Fisher Scientific, Waltham, MA, USA. Whatman filter paper No. 1 with a diameter of 110 mm (Whatman International Ltd., Maidstone, UK) was used throughout the research. All the chemicals used in this study were of analytical grade.

### 4.2. Surimi Gel Making

The surimi production protocol was adapted from a method established by Zhang et al. [27]. The fresh SC fillets were split into five groups: one fresh group and four groups subjected to frozen storage for 1, 2, 3, and 6 months. Fresh SC fillets were cut into 4–5 cm segments, and manually deboned and deskinned. The prepared fish segments underwent three washing cycles with cold deionized water (5 °C) to eliminate residual blood and soluble remains. Excess surface water was removed using gauze, resulting in a final moisture content of 78–80%. Then the prepared fish segments were minced using a meat blender (Oster 21304893 Cup Mini Food Chopper, Sunbeam Products, Inc., Boca Raton, FL, USA) maintained below 10 °C in an ice bath for 2 min to form a uniform mince. Then the mince was screened through a 16-mesh screen on ice to remove the fascia and small bone particles. The screened mince was divided half and half: one for control samples, and the other was supplemented with TG. Based on the findings of our previous study that low dosage of TG had a strong effect to enhance FSC surimi [19], 0.1 wt% TG was applied. The control group was mixed with 2.5 wt% NaCl, while the TG group was mixed with 0.1 wt% TG and 2.5 wt% NaCl. Both were blended under the chilled condition for 1 min. A small portion of the paste was stored at 5 °C for further Rheology tests, while the remainder was stuffed into 20 × 20 mm cylindrical casings, which were sealed and subjected to a two-step heat treatment: first at 35 °C for 2 h, followed by 90 °C for 20 min. This heating process was selected to optimize TG enzyme activity and gel formation [27]. Each group has three replicated samples. After heating, the gels were removed from their casings and stored at 5 °C until analysis. The frozen-stored SC fillets were thawed at 5 °C for 24 h once they reached the targeted frozen time, and followed the same protocol as the fresh group to make samples.

### 4.3. Gel Strength Test

Gel strength was determined as the product of breaking force (N) and deformation distance (m) at the point of break. These parameters were measured using a TA.XT Plus Texture Analyzer (Texture Technologies Corp., Surrey, UK) fitted with a P/5 probe, following a method adapted from Zhang et al. [27]. The instrument settings were: a pre-test speed of 2.0 mm/s, test speed of 1.0 mm/s, post-test speed of 10 mm/s, a trigger force of 10 g, and a 50% compression ratio.

### 4.4. Texture Profile Analysis (TPA) Test

The textural properties of the surimi gels, including hardness, cohesiveness, springiness, resilience, and chewiness, were measured using a Texture Profile Analysis (TPA). The analysis was also conducted on a TA.XT Plus Texture Analyzer (Texture Technologies Corp., Surrey, UK) according to a procedure adapted from Zhang et al. [27]. A cylindrical probe (P/50) was used to compress the samples to 30% of their original height at a test speed of 1.0 mm/s, with pre-test and post-test speeds set to 2.0 mm/s and 10 mm/s, respectively. A trigger force of 10 g was applied to initiate the test. The TPA parameters were automatically calculated from the resulting force-time curve by Exponent (Version 6.1, Stable Micro Systems, Surrey, UK).

### 4.5. Cooking Loss and Expressible Moisture

The method to find cooking loss was modified from Zhu et al. [31]. The mass of each surimi sample before heating gelation was recorded as m0. After heating, the surface water of surimi gel was wiped with filter paper, and the surimi mass was recorded the mass m1. The cooking loss was calculated as follows:

(1) Cooking loss = (m0 − m1)/m0 × 100%

The expressible moisture was determined by modifying Petcharat’s group [36]. The gel samples were sectioned into 5 mm-thick discs and weighed (X). Each disc was then sandwiched between two layers of filter paper below and two layers above. A constant 5 kg weight was applied to the stack for two minutes. Following this compression, the sample was reweighed (Y). The expressible moisture was calculated as a percentage of the initial sample weight using the following equation:

(2) Expressible moisture = (X − Y)/X × 100%.

### 4.6. Rheological Test

The viscoelastic properties of the SC paste were characterized using small-amplitude oscillatory shear (SAOS) testing on a Discovery HR-2 Rheometer (TA Instruments, New Castle, DE, USA). Following the method of Xie et al. [33], a frequency sweep was performed at a fixed strain of 1% across a frequency range of 0.1 to 100 Hz. Sample discs (1 mm thick) were tested using a 40 mm diameter parallel plate geometry. The storage modulus (G′), loss modulus (G″), and the tan б were recorded in response to the frequency change.

The thixotropic behavior of the SC paste was evaluated using a hysteresis loop test, following a method adapted from Xie et al. [33]. The test involved increasing the shear rate from 0.01 to 100 s^−1^ over a period of 180 s (upward curve), followed by a decrease to 0.01 s^−1^ over the same duration (downward curve). Sample discs (1 mm thick) were tested using a 40 mm diameter parallel plate geometry. Shear stress and viscosity were recorded throughout the test to characterize the time-dependent rheological properties of each sample.

### 4.7. Multiple Machine Learning Models Training

To identify the optimal predictive model for synthetic data generation, numerous regression algorithms were evaluated in Jupyter Notebook (Version 7.4, Project Jupyter, hosted by LF Charities, Inc., San Francisco, CA, USA) with Python (Version 3.11, Python Software Foundation, Wilmington, DE, USA). These algorithms included Linear Regression, Polynomial Regression, Ridge Regression, Support Vector Regression (SVR), Decision Tree, Random Forest, Gradient Boosting, Bayesian Ridge, Automatic Relevance Determination (ARD), Multilayer Perceptron (MLP), and Extreme Gradient Boosting (XGB). The hyperparameters of these models were set as default values because they were usually chosen by library developers to work reasonably well across a variety of datasets and provided a baseline performance. Although changing hyperparameters would improve the model performance sometimes, it increases the complexity of the method, which is not tech-friendly for non-experts.

Each model was trained on our previously published experimental dataset for gel strength, hardness, and chewiness of FSC surimi [19]. The data were generated using a Box–Behnken design (BBD) to investigate the impact of combinations of additives with different dosages (0.1, 0.5, 1.0 wt% MMF; 0.1, 0.5, 1.0 wt%TG; and 1.0, 3.0, 5.0 wt% CLG) on FSC surimi properties. The BBD comprised 15 combinations, each with 4 repetitions, resulting in a total of 60 data points. For model development, this dataset was randomly separated into training (42) and validation (18) groups. The model performance was assessed by comparing the predicted values (ŷ_i_) and actual values (y_i_) using the coefficient of determination (R^2^). The equation of R^2^ calculation was as follows, where n is the number of data points [37]:

(3) $$R^{2}=1-\frac{\sum_{i=1}^{n}{\left(y_{i}-\hat{y_{i}}\right)}^{2}}{\sum_{i=1}^{n}{\left(y_{i}-\bar{y}\right)}^{2}},\;where\;\bar{y}=\frac{1}{n}\sum_{i=1}^{n}y_{i}.$$

The R^2^ directly reflects the proportion of variance in the response explained by the model and allows consistent comparison across gel strength, hardness, and chewiness. Error-based metrics such as RMSE or SMAPE are scale-dependent and less comparable across characteristics, while R^2^ provides an intuitive, unit-free measure [37]. The best-performing model, defined by the highest mean of R^2^ value, was subsequently employed in the data generation model.

### 4.8. Synthetic Data Generation and Validation

The synthetic data generation method was modified from Zhang’s group [25], who used the multi-distribution global trend diffusion (MD-MTD) method to generate spectral-like data, which is too complicated for our small, tabulated dataset. For a small dataset, kernel density estimation (KDE) is a common method to generate new data by approximating the underlying probability distribution [26]. The main steps of the data generation method were shown in Figure 7 and could be described as follows:

1. Initial Model Training: A Random Forest Regressor (RFR) was trained on the original, limited dataset of input-output pairs (x_original and y_original);
2. Modeling the Input and Output Distribution: A Kernel Density Estimation model was independently fitted to both the input and output values of the original data, and generated new input and output values (x_new and y_new) that resided in regions supported by the original data;
3. Finding Absolute Error: The trained RFR was used to predict the output for x_new to gain y_pred. The absolute error was calculated between the y_pred and y_new for the same x_new;
4. Defining Threshold: The validation threshold (τ) was defined as a fraction of the original output’s standard deviation (SD) as follows, where the fit value was adjustable to reach a desired bias between y_pred and y_new:
  (4) τ = fit value × SD;

5. Validation Loop: If absolute error ≤ τ, the x_new and y_new were deemed consistent with the RFR’s understanding of the input-output relationship and were accepted. If absolute error > τ, the y_new was rejected, as the discrepancy between the KDE-generated output and the RFR-predicted output is too large, and returned to step 2;
6. Termination Condition: The loop iterates, batch-wise, until the number of accepted samples reaches the exact target.

Following the SDDML method with a fit value of 0.003, 240 synthetic data points were generated for gel strength, hardness, and chewiness. The 240 generated data were used as a training group to train the Random Forest model, and the 60 original data were used as a testing group. The R^2^ of the model was computed to validate the representativeness of the generated data.

To evaluate the model’s accuracy, a combination of original and synthetic points was split into training (240) and validation (60) sets. The distribution of R^2^ scores for gel strength, hardness, and chewiness was analyzed.

For a more direct test, 18 original data points were held out as a final testing set. The model was then trained on the remaining 42 original data points, augmented with different synthetic data sizes (0, 60, 120, 240, 600, 1200). The average determination coefficients (R^2^) out of 100 repetitions (each repetition with a randomly selected 18 data point testing set) were compared.

### 4.9. Data Analysis and Model Program

JMP (Version 17.2, SAS Institute Inc., Cary, NC, USA) was used for Tukey’s honestly significant difference (Tukey’s HSD) test to determine the significant differences among datasets. The *p*-value was set to *p* < 0.05.

Jupyter Notebook (Version 7.4, Project Jupyter, USA) with Python (Version 3.11, Python Software Foundation, Wilmington, DE, USA) was used to plot figures and build models. A template of an SDDML Jupyter Notebook file (accessed on 27 August 2025), including the program described in Section 4.7 and Section 4.8, is available below: https://github.com/JingyiY99/SDDML-Temp (accessed on 27 August 2025).

## Figures and Tables

**Table 1:** Springiness, cohesiveness, and resilience results of control FSC surimi samples and 0.1 wt% TG-added FSC surimi samples along with frozen storage time.

Storage Time (Months)	Treatment	Springiness (Mean ± SEM, *n* = 3)	Cohesiveness (Mean ± SEM, *n* = 3)	Resilience (Mean ± SEM, *n* = 3)
0	Control	0.901 ± 0.003 ^A^	0.773 ± 0.001 ^C^	0.405 ± 0.002 ^D^
	0.1 wt% TG	0.881 ± 0.014 ^A^	0.794 ± 0.003 ^BC^	0.440 ± 0.003 ^BC^
1	Control	0.881 ± 0.013 ^A^	0.777 ± 0.007 ^C^	0.434 ± 0.007 ^BC^
	0.1 wt% TG	0.937 ± 0.012 ^A^	0.815 ± 0.009 ^AB^	0.482 ± 0.007 ^A^
2	Control	0.913 ± 0.004 ^A^	0.775 ± 0.003 ^C^	0.443 ± 0.003 ^B^
	0.1 wt% TG	0.939 ± 0.017 ^A^	0.825 ± 0.003 ^A^	0.496 ± 0.006 ^A^
3	Control	0.907 ± 0.003 ^A^	0.793 ± 0.003 ^BC^	0.430 ± 0.001 ^BCD^
	0.1 wt% TG	0.917 ± 0.005 ^A^	0.823 ± 0.003 ^A^	0.497 ± 0.003 ^A^
6	Control	0.903 ± 0.004 ^A^	0.773 ± 0.003 ^C^	0.417 ± 0.003 ^CD^
	0.1 wt% TG	0.902 ± 0.007 ^A^	0.817 ± 0.003 ^AB^	0.487 ± 0.003 ^A^

Different uppercase letters in a column indicate significant differences (*p* < 0.05).

**Table 2:** Determination coefficient (R^2^) on the validation set for different ML methods based on experimental data with MMF, TG, and CLG as additives to improve FSC surimi gel * [19].

Quality Attributes (QAs)	Performance Summary	ML Methods										
		Linear Regression	Polynomial Regression **	Ridge Regression **	SVR	Decision Tree	Random Forest	Gradient Boosting	Bayesian Ridge **	ARD	MLP	XGB
Gel Strength	Mean± SD	0.272 ± 0.325	0.369 ± 0.340	0.402 ± 0.248	0.304 ± 0.302	0.403 ± 0.265	0.464± 0.218	0.406 ± 0.264	0.406 ± 0.264	0.410 ± 0.264	0.005 ± 0.618	0.409 ± 0.262
	Best Case	0.620	0.824	0.724	0.732	0.825	0.804	0.824	0.729	0.734	0.728	0.825
Hardness	Mean± SD	0.054 ± 0.311	0.492 ± 0.297	0.502 ± 0.256	0.493 ± 0.273	0.588 ± 0.252	0.605± 0.220	0.588 ± 0.255	0.508 ± 0.262	0.526 ± 0.247	0.115 ± 0.804	0.586 ± 0.259
	Best Case	0.467	0.837	0.817	0.885	0.862	0.889	0.862	0.826	0.817	0.885	0.873
Chewiness	Mean± SD	−0.173 ± 0.220	0.199 ± 0.409	0.270 ± 0.272	0.577 ± 0.263	0.595 ± 0.278	0.617± 0.196	0.606 ± 0.240	0.266 ± 0.288	0.260 ± 0.425	−0.120 ± 0.981	0.584 ± 0.327
	Best Case	0.122	0.595	0.642	0.897	0.919	0.906	0.919	0.642	0.641	0.890	0.919

* This dataset contained 60 data points, randomly separated into training (42) and validation (18) groups. The random state of the model is tested from 0 to 99. ** The polynomial degree used for Polynomial Regression, Ridge Regression, Bayesian Ridge, and ARD was 2.

**Table 3:** Mean determination coefficient (R^2^) ± SD on the testing set for different sizes of synthetic datasets using the SDDML model.

QAs	Size of Synthetic Dataset Added to Experimental Data (42) in Training					
	0	60	120	240	600	1200
Gel Strength	0.464 ± 0.218 ^A^	0.470 ± 0.209 ^A^	0.471 ± 0.216 ^A^	0.476 ± 0.206 ^A^	0.481 ± 0.215 ^A^	0.485 ± 0.208 ^A^
Hardness	0.605 ± 0.220 ^A^	0.646 ± 0.193 ^B^	0.645 ± 0.195 ^B^	0.648 ± 0.195 ^B^	0.646 ± 0.197 ^B^	0.646 ± 0.196 ^B^
Chewiness	0.617 ± 0.196 ^A^	0.670 ± 0.137 ^B^	0.671 ± 0.143 ^B^	0.679 ± 0.136 ^B^	0.679 ± 0.134 ^B^	0.678 ± 0.137 ^B^

Different uppercase letters in a row indicate significant differences (*p* < 0.05).

## Data Availability

The data presented in this study are available upon request from the corresponding author.

## Abbreviations

The following abbreviations are used in this manuscript:

SC	Silver Carp
FSC	Frozen Silver Carp
SDDML	Synthetic Data-Driven Machine Learning
WHC	Water-holding Capacity
MMF	Manufactured Microfiber
TG	Transglutaminase
CLG	Collagen
RSM	Response-Surface Modeling
ML	Machine Learning
SVR	Support Vector Regression
ARD	Automatic Relevance Determination for Bayesian Ridge
MLP	Multilayer Perceptron
XGB	Extreme Gradient Boosting
KDE	Kernel Density Estimation
TPA	Texture Profile Analysis
SAOS	Small-amplitude Oscillatory Shear
EM	Expressible Moisture
MD-MTD	Multi-distribution Global Trend Diffusion
RFR	Random Forest Regressor
SD	Standard Deviation
HSD	Honestly Significant Difference
QAs	Quality Attributes
SEM	Standard Error of the Mean
